# Prostate Cancer Progression Modeling Provides Insight into Dynamic Molecular Changes Associated with Progressive Disease States

**DOI:** 10.1158/2767-9764.CRC-24-0210

**Published:** 2024-10-24

**Authors:** Runpu Chen, Li Tang, Thomas Melendy, Le Yang, Steve Goodison, Yijun Sun

**Affiliations:** 1 Department of Microbiology and Immunology, University at Buffalo, State University of New York, Buffalo, New York.; 2 Department of Computer Science and Engineering, University at Buffalo, State University of New York, Buffalo, New York.; 3 Department of Cancer Prevention and Control, Roswell Park Comprehensive Cancer Center, Buffalo, New York.; 4 Department of Quantitative Health Sciences, Mayo Clinic, Jacksonville, Florida.

## Abstract

Prostate cancer is a significant health concern and the most commonly diagnosed cancer in men worldwide. Understanding the complex process of prostate tumor evolution and progression is crucial for improved diagnosis, treatments, and patient outcomes. Previous studies have focused on unraveling the dynamics of prostate cancer evolution using phylogenetic or lineage analysis approaches. However, those approaches have limitations in capturing the complete disease process or incorporating genomic and transcriptomic variations comprehensively. In this study, we applied a novel computational approach to derive a prostate cancer progression model using multidimensional data from 497 prostate tumor samples and 52 tumor-adjacent normal samples obtained from The Cancer Genome Atlas study. The model was validated using data from an independent cohort of 545 primary tumor samples. By integrating transcriptomic and genomic data, our model provides a comprehensive view of prostate tumor progression, identifies crucial signaling pathways and genetic events, and uncovers distinct transcription signatures associated with disease progression. Our findings have significant implications for cancer research and hold promise for guiding personalized treatment strategies in prostate cancer.

**Significance:**

We developed and validated a progression model of prostate cancer using >1,000 tumor and normal tissue samples. The model provided a comprehensive view of prostate tumor evolution and progression.

## Introduction

Prostate cancer is a significant health burden, representing the second most commonly diagnosed cancer in men in the United States and the most common worldwide (1–3). It is a clinically heterogeneous disease, with diverse phenotypes being driven by distinct patterns of genetic variations and pathway regulation. An understanding of the molecular patterns associated with specific disease states, and importantly, how these patterns change throughout disease progression could provide crucial insights for the development of improved patient evaluation and clinical management.

Several attempts have been made previously to study the dynamics of prostate cancer progression (4–10), which can be broadly classified into two categories. The studies in the first category utilized phylogenetic analysis (4–7), by comparing mutation or copy number variation (CNV) profiles of a small number of evolutionary-related tumor samples (e.g., those collected from the same patient before and after surgery or from different regions of the same tumor). Although phylogenetic analysis confirms the cancer evolution theory, constructed models reflect only the evolutionary histories of individual tumors at the time of sampling and cannot be generalized to other patients because even tumors of the same phenotype can have completely different mutational and CNV profiles. Therefore, phylogenetic analysis cannot establish a unified evolutionary model for a disease. The studies in the second category utilized lineage analysis. In a notable study (8), researchers obtained single-cell RNA sequencing data from 13 prostate tumor samples to describe potential disease progression patterns based on the differentiation of epithelial cell lineages and the status of the tumor microenvironment. However, as single-cell studies are necessarily limited by cost to relatively small sample sizes, it is difficult to capture the whole spectrum of progression processes of the disease (9). Moreover, the difficulty in obtaining single-cell multiomics data restricts the joint analyses of genetic and transcriptomic variations in such studies. Another study (10) investigated the order and mutual exclusivity between important genetic events and proposed a progression model composed of two tumor lineages, namely *ERG*–*PTEN* and *SPOP*–*CHD1*. However, the study considered only a few preselected genetic events in the lineage construction, and consequently, the resulting progression model is of relatively low resolution.

In our previous work (11), we have proposed a novel computational approach for cancer progression modeling that can overcome the aforementioned limitations by utilizing large-scale static sample cohorts. The approach leverages the notion that each tumor sample represents a snapshot of a disease process, and by analyzing a sufficiently large cohort of samples, we can reconstruct the entire progression process computationally (11). We have successfully applied the approach to breast cancer datasets and constructed a novel bifurcating progression model. The model was validated using independent datasets (11) and further confirmed through the examination of matched primary and metastatic breast cancer tumors (12). Our previous work demonstrated the feasibility of elucidating disease progression patterns using static sample data and provided one of the first working models of breast cancer progression.

In this study, we performed a large-scale analysis by applying a similar computational approach to a dataset obtained from The Cancer Genome Atlas (TCGA) program and established a progression model of prostate cancer. By using the developed model as a contextual foundation, we conducted a joint transcriptomic and genetic analysis to detect putative cancer driver genes, key signaling pathways, and genetic events that occur during disease progression. The application of the approach to a large cohort (∼500 patients) enabled the establishment of a comprehensive and high-resolution progression model that reflects the spectrum of prostate cancer. Furthermore, by ordering tumor sample data on the identified progression paths, we were able to identify specific transcription signatures associated with disease progression and to classify tumor samples into distinct disease progression states. By comparing the detected states, we elucidated the timing of key driver events in disease progression. The derivation of a high-resolution prostate cancer progression model provides the opportunity to glean novel insights into the disease that can be leveraged for cancer research and clinical benefit.

## Materials and Methods

### Bioinformatics pipeline for prostate cancer progression modeling

The bioinformatics pipeline consists of three main components. First, we performed a feature selection analysis to identify disease-related genes. Then, by using the selected genes, we constructed a principal tree to mathematically describe the general trend of the data. Finally, by using the principal tree as a backbone, we built a cancer progression model.

#### Feature selection to identify disease-related genes

Because only a small fraction of genes are likely to be involved in cancer development, the first step toward progression modeling is to identify disease-related genes. This is a critical yet difficult step, forming the basis for all downstream analyses. We formulated it as a feature selection problem for multiclass supervised learning. Specifically, we used Gleason scores (GS) as class labels to guide the selection of relevant genes. Following the suggestion given by (13, 14), we partitioned tumor samples into four different risk groups: GS ≤ 6, GS = 3 + 4, GS = 4 + 3, GS = 8, and GS ≥ 9. For the purpose of this study, we employed the LOGO algorithm (15) for the feature selection analysis. The LOGO algorithm is one of the most competitive feature selection methods derived to date, with excellent accuracy and computational efficiency. The basic idea is to decompose a complex nonlinear classification problem into a set of locally linear ones and then learn feature relevance globally within a large-margin framework (15). It is able to handle an arbitrarily complicated classification problem without making any assumption on the underlying data distribution and therefore is well suited for our purpose. . Below, we give a brief description of the algorithm.

Let $\{(x_{n},y_{n}){\}}_{n=1}^{N}$ be the dataset, in which **x**_*n*_ is the *n*th sample and *y*_*n*_ is the corresponding class label. We associate each sample with a feature weight vector **w** ≥ 0, in which the magnitude of each element represents the relevance of the corresponding feature or gene. Our goal is to find a subspace specified by **w** in which test samples can be correctly classified. We start by defining the margin of each sample. Given sample **x**_*n*_, we find its two nearest neighbors in the same class [denoted as nearest-hit, NH(**x**_*n*_)] and in a different class [denoted as nearest-miss, NM(**x**_*n*_)], respectively. The margin of **x**_*n*_ with respect to **w** is defined as $\rho_{n}(w)=d(x_{n},\mathrm{NM}(x_{n})|w)-d(x_{n},\mathrm{NH}(x_{n})|w)$, in which *d*(·) is a distance function. In this study, we used the Manhattan distance. Therefore, the margin can be rewritten as $\rho_{n}(w)=w^{T}(|x_{n}-\mathrm{NM}(x_{n})|-|x_{n}-\mathrm{NH}(x_{n})|)≜w^{T}z_{n}$. By the large-margin theory (16), a classifier generalizes well on unseen test samples if a margin-based loss function is minimized. We use the logistic loss function and obtain the following optimization problem:

$$\min_{w}\sum_{n=1}^{N}\log\left(1+\exp(-w^{T}z_{n})\right)+\lambda \|w{\|}_{1}\text{, subject to }w\geq 0,$$ (1)

in which we impose an $\ell_{1}$-norm constraint on *w* to obtain a sparse solution, and *λ* is a regularization parameter that can be tuned through cross-validation. An issue with the above formulation is that the nearest neighbors of a given sample are unknown before learning. To address the issue, we adopt an expectation–maximization strategy, in which the nearest neighbors of a sample are treated as hidden variables, and the margin is estimated by averaging out hidden variables. Specifically, we first make a guess on **w**. Then, we calculate pairwise distances and estimate the probability of each sample being the nearest miss or hit of another sample through kernel learning. Finally, we update **w** by solving the above optimization problem. The process iterates until convergence. To reduce the number of parameters to be tuned, in this study, we employed the Epanechnikov kernel (17) and set the kernel size to 5. Once we obtained a feature weight vector, we normalized the vector by dividing each element by the maximum value of the vector and retained the genes with a feature weight larger than 1e-2 for downstream analyses.

#### Principal curve learning to delineate transcriptional dynamics

After we detected disease-related genes, the next challenge was to build a model to mathematically describe disease dynamics. To this end, principal curve fitting methods were used. Formally, a principal curve is a nonlinear generalization of the first principal component line passing through data cloud (18). In the last decade, a dozen methods have been developed for principal curve fitting (18, 19). However, they are generally limited to learn a curve that is embedded in a low-dimensional space and does not intersect itself (18, 19), which is quite restrictive for real applications. We recently developed a new graphic model–based method, referred to as DDRTree, to learn a tree-like structure from data that addresses some limitations of prior work (20, 21).

Let $\{x_{i}{\}}_{i=1}^{I}\in X\subset {\mathbb{R}}^{D}$ be the input data, and G={V,E} be an undirected graph in which $V=\{V_{i}{\}}_{i=1}^{I}$ is a vertex set, and E is an edge set. We assume that graph G lies in a latent space $R^{d}$ with *d*≪*D* and associates every vertex *V*_*i*_ with a latent point $z_{i}\in Ƶ\subset {\mathbb{R}}^{d}$. Our goal is to learn a transformation function $f_{G}$ that maps data points in the intrinsic space Ƶ to the input space X. To this end, we further assume that the observed data are generated through a random process. Specifically, we first sample a data point $z_{i}\in Ƶ$, then corrupt **z**_*i*_ with some random noise to generate **y**_*i*_, and finally map **y**_*i*_ to the input space. For the purpose of the study, we use a linear mapping function $f(y_{i})=Wy_{i}$ with **$W^{T}W=I$** as the transformation function and set G to a minimum spanning tree (22) to represent disease dynamics, in which the length of an edge is defined as the squared Euclidean distance between two adjacent latent points. By combining the above considerations, we obtain the following formulation:

$$\min_{W,B,R,Ƶ,Y}\sum_{i=1}^{I}\|x_{i}-Wy_{i}{\|}^{2}+\gamma \sum_{i,j=1}^{I}r_{i,j}\left(\|y_{i}-z_{j}{\|}^{2}+\sigma \log r_{i,j}\right)+\frac{\lambda }{2}\sum_{i,j=1}^{I}b_{i,j}\|z_{i}-z_{j}{\|}^{2}\text{, subject to }W^{T}W=I,\sum_{i=1}^{I}r_{i,j}=1,r_{i,j}\in \left[0,1\right],\forall i,j,$$

in which the first term is the reconstruction error, the second term is the representation error, the third term is the total curve length of a principal tree in the $\ell_{2}$-norm form, {*b*_*i,j*_} is constrained to be a feasible solution of a minimum spanning tree that takes a value of 1 if $(V_{i},V_{j})\in E$ and 0 otherwise, *r*_*i,j*_ is the probability of assigning **y**_*i*_ to **z**_*j*_, and *σ* is a parameter for soft assignment using negative entropy regularization. The above optimization problem can be solved by using alternating structure optimization (20). To avoid tuning too many parameters, following the work in (20), we set *γ* = 5 and estimated *λ* and *σ* using the elbow method (23).

#### Construction of a progression model

We used the principal tree result to construct a progression model, to extract progression paths, to calculate progression distances of tumor samples, and to derive disease progression states. By construction, the principal tree is a graphical model, in which a leaf node (i.e., terminus) represents a sample that is connected with another sample. We designated the leaf node closest to the center of the normal samples as the root vertex to represent the origin of disease progression. We extracted as a progression path the shortest path from the root vertex to a non-root leaf node of the principal tree. To calculate progression distances of tumor samples, we first projected each sample back onto the principal tree. Here, the projection of a sample is defined as a point on the principal tree that is the closet to the sample. Then, we calculated the progression distance of a sample as the geodesic distance from the root vertex to the projection of the sample. In addition, we used the branching points of the tree to divide the principal tree into segments. Then, based on the projection of each sample, we determined which segment it falls into, leading to the identification of the seven progression states.

### Running sum enrichment analysis

We conducted a running sum enrichment analysis to study the association between a discrete variable and a progression path. For a discrete variable, we calculated an enrichment score reflecting the degree to which one of the variable’s states (+1 or −1) was enriched at the beginning or end of the path. Let the number of the samples in the +1 or −1 state be *N*_*+*_ and *N*_*−*_, respectively. The enrichment score was computed by walking down the path, increasing a running sum statistic by 1/*N*_+_ for +1 and decreasing it by 1/*N*_−_ for −1. The score is the maximum deviation from zero in this walk. A positive score indicates that +1 is enriched at the beginning (and that −1 is enriched at the end), whereas a negative score indicates that +1 is enriched at the end (and that −1 is enriched at the beginning). The statistical significance of the score was estimated by permuting the variable distribution along the path and recalculating the enrichment score. The *P* value was then calculated by the frequency of permuted scores equal to or more extreme than the observed score. To ensure the statistical power, we used 10,000 permutations to determine the *P* values.

### Transcriptional analysis to characterize transcriptome dynamics

To characterize the transcriptome dynamics associated with disease progression, we performed a transcriptional analysis to identify genes whose expression levels changed significantly along progression paths. To this end, a hypothesis test was performed. Specifically, given a gene, under a null model, we assumed that the gene expression level does not change along a progression path and modeled the expression level as a constant function of the progression distance. In contrary, under an alternative model, we assumed that the gene expression varies as a nonlinear function of the progression distance. For the purpose of this study, we used a natural spline function to describe the expression pattern. Following the suggestion given by (24), the degree of freedom (df) of a natural spline function was set to be 3. Then, we computed a *P* value by performing the likelihood–ratio test to compare the goodness-of-fit of the two models (25). Finally, we performed multiple testing correction and estimated FDRs using the Benjamini–Hochberg (BH) method. We retained genes with FDR ≤ 1e–4 for downstream analyses.

After we identified significantly varying genes for each path, we performed a clustering analysis to group the genes into up- and downregulated gene modules. Specifically, we first fitted a natural spline function (df = 3) to the expression data of each gene to generate a gene curve, and then clustered the generated curves into two modules by using the *k*-medoids algorithm (26). In the clustering analysis, the distance between two gene curves **x** and **y** was defined as *d*_**x,y**_ = 1−*ρ*_**x,y**_/2, in which *ρ*_**x,y**_ is the Pearson correlation between **x** and **y**. We found that one cluster contained all the genes with expression levels exhibiting an upward trend along a progression path (thus denoted as upregulated module), whereas another cluster contained all the genes with expression levels showing a downward trend (thus denoted as downregulated module; Supplementary Fig. S1).

### Transcriptional analysis to identify path-dependent progression signatures

We performed a transcriptional analysis to identify genes with distinct expression patterns along different progression paths. To this end, the branch expression analysis model (BEAM; ref. 27) was employed. Specifically, given a gene, under a null model, we assumed that the expression pattern of the gene was the same for two paths. Thus, we pooled the samples assigned to the two paths together and fitted a single natural spline curve (df = 3) to describe the gene expression pattern. On the contrary, under an alternative model, we assumed that the expression patterns were path-dependent. Therefore, we fitted two natural spline curves (df = 3) to the samples from the two paths. We compared the goodness-of-fit of the two models using the likelihood-ratio test (25) and generated a *P* value. Then, we estimated FDRs using the BH method and selected genes with FDR ≤ 1e–4. We employed the *k*-medoids algorithm (26) to cluster the identified genes into modules. As with the previous analysis, the Pearson distance was used as the distance metric and the optimal number of clusters was selected from {2, ⋯, 8} by using the maximum silhouette rule (28).

### Gene set enrichment analysis

We performed a gene set enrichment analysis (29, 30) on the identified gene modules to evaluate whether the genes in a biological pathway are overrepresented or significantly enriched in a gene module. For the purpose of the study, we used the pathways obtained from the Kyoto Encyclopedia of Genes and Genomes (KEGG, RRID: SCR_012773) database. Specifically, given a set of genes from a KEGG pathway, we performed the right-tailed Fisher’s exact test to compare the number of genes from the set that are also presented in an identified gene module with the expected number obtained by random chance. After we obtained *P* values through the test, we applied the BH method to estimate FDRs and selected pathways with FDR ≤ 1e–2 for downstream analyses.

### Validation experiment on an independent dataset

The dataset was downloaded from the Gene Expression Omnibus (GEO, RRID: SCR_005012) Repository (GEO accession number: GSE46691). Unlike the TCGA data, the GSE data provided only the combined GS. We partitioned the data into four groups (GS ≤ 6, GS = 7, GS = 8, and GS ≥ 9). As with the analysis performed on the TCGA data, we employed the LOGO algorithm (15) for identifying disease-related genes and the DDRTree algorithm (20) for principal curve fitting. To extract disease progression paths, we first inferred the *ERG* fusion status based on the *ERG* gene expression levels. Then, we determined as the disease origin the leaf node that is closest to the *ERG* fusion–positive and –negative mixture group. Starting from the disease origin, we extracted three progression paths by designating the remainder three leaf nodes as progression termini. To align the three extracted progression paths with the four paths identified in the TCGA model, we developed a *k*-nearest neighbors classifier based on the disease-related genes identified in the TCGA data and classified each sample in the GSE data as one of the seven states identified in the TCGA data. Specifically, *k* was set to 5, and the Pearson distance was used as the distance metric.

### Data availability

The data and methods used in the study are summarized in Supplementary Table S1, which provides the citations of the relevant references and links to repository to access data and/or code.

## Results

### Overview of the study

We first constructed a progression model of prostate cancer using the gene expression data obtained from the TCGA study (31). To demonstrate the validity of the constructed model, we performed a series of analysis by mapping clinical and molecular variables with the implication of cancer progression onto the identified progression paths. Then, we conducted a trajectory analysis to identify transcriptional signatures associated with the modeled progression paths. We also mapped mutation and copy number data onto the constructed model to identify key genetic events occurring at different progression states. To further investigate the validity of the constructed model, we performed a progression modeling analysis on an independent prostate cancer dataset.

### Constructing a prostate cancer progression model using TCGA data

The TCGA dataset contains the expression levels of 20,531 genes from 497 prostate tumor samples and 52 normal tissue samples (31). Before the downstream analyses, the data were log-transformed. Because only a small fraction of genes are likely to be involved in cancer development, the first step toward progression modeling is to identify disease-related genes. To this end, we formulated it as a feature selection problem for supervised learning and used as class labels the summary GS (sum of primary and secondary Gleason grade) due to its important role in determining the aggressiveness and prognosis of prostate tumors (see “Materials and Methods” for details; refs. 14, 32). A parsed set of 43 genes were identified as being representative of disease-related genes (Supplementary Table S2). By using the expression data of the selected genes, we performed progression modeling analysis to construct a principal tree by using the DDRTree algorithm (20, 21) to mathematically describe disease dynamics (“Materials and Methods”).

**Figure 1:**
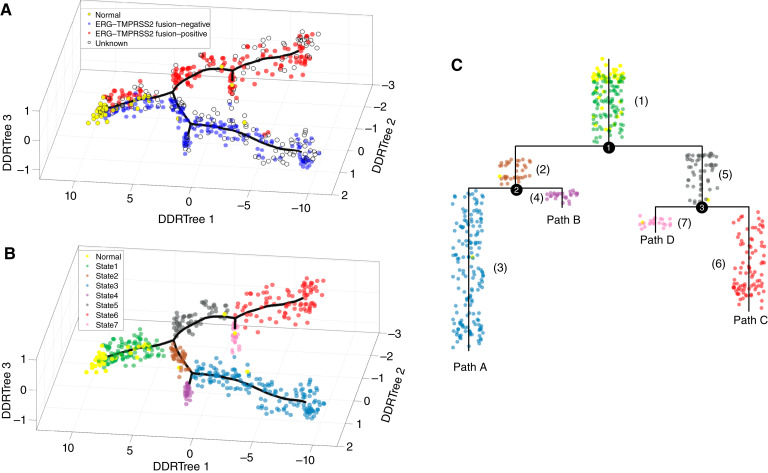
Progression model of prostate cancer constructed by using TCGA data. **A,** Visualization analysis showed a general trend of data distribution of normal and tumor samples. Each tumor sample was color-coded by its *TMPRSS2*–*ERG* fusion status. The black line is the constructed principal tree, and the three axes represent the leading components generated by the DDRTree algorithm (denoted as DDRTree 1–3). **B,** Seven progression states were identified by partitioning the samples on the data manifold. State 1 is close to the origin of disease progression, and states 3, 4, 6, and 7 are the termini of the progression paths. **C,** Four progression paths were identified, referred to as paths A–D, respectively. The solid numbers are the major branch points of the constructed tree, and the numbers in brackets are the identified disease progression states.

Figure 1A shows the distribution of the tumor and normal samples in the space spanned by the three leading components (denoted as DDRTree 1–3) generated using the DDRTree algorithm. The black line is the constructed principal tree. Previous studies have established that *TMPRSS2*–*ERG* fusion is the most frequent genomic alteration in prostate cancer, present in ∼50% of cases, and that *TMPRSS2*–*ERG* fusion is an early event in disease progression (10, 33, 34). Accordingly, we color-coded each tumor sample by its *TMPRSS2*–*ERG* fusion status (164 tumor samples had unknown status). By utilizing the topologic characteristics of the data manifold, we partitioned the tumor samples into seven progression states (Fig. 1B; Supplementary Table S3). We designated the leaf node of the principal tree that is closest to the center of the normal samples as the starting point of disease progression and extracted four progression paths, referred to as paths A–D, respectively (Fig. 1C; Supplementary Table S4). Our analysis revealed a linear, bifurcating progression pattern, originating from the normal samples, gradually shifting to state 1 with mixed *ERG* fusion–positive and –negative tumor samples, and diverging to two distinct trajectories to malignancy defined by the presence (referred to as *ERG* fusion–positive) or absence (*ERG* fusion–negative) of the *ERG* gene fusion (*P* < 3.8e–35, binomial test). Significant side branches are also evident for the *ERG* fusion–positive branch (i.e., state 7) and the *ERG* fusion–negative branch (i.e., state 4). Our result is consistent with previous findings that support the independent development of *ERG* fusion–positive and –negative prostate cancer from the normal prostate gland (10, 33).

### Validating the constructed model using clinical and genetic variables

To evaluate the validity of the constructed model, we performed a series of analyses by mapping onto the model the clinical and molecular variables with the implication of cancer progression. The rationale is that if the constructed model is valid, we would expect a clinical or genetic variable associated with cancer development to be significantly correlated with the modeled progression paths and malignant tumor samples (e.g., high-grade tumors) to be enriched toward the termini of the progression paths.

**Figure 2:**
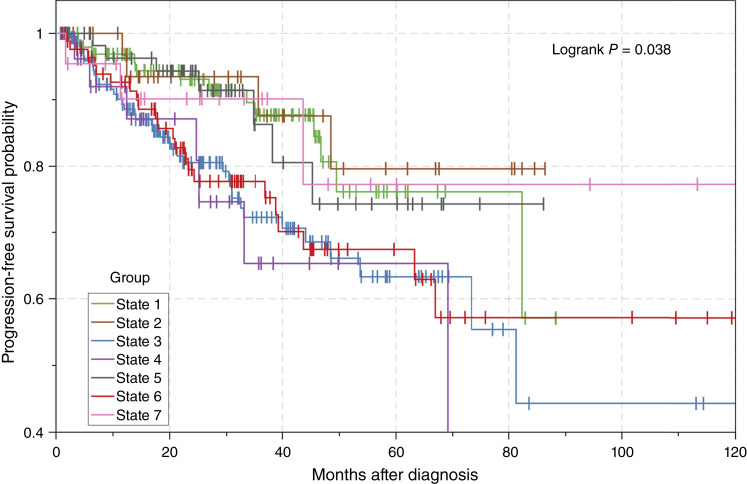
Progression-free survival functions of seven identified tumor states.

We first mapped the clinical progression-free survival data (extracted from the TCGA data) back onto the model and performed a survival analysis of the seven identified tumor states. Here, the progression-free interval is defined as the period from the date of diagnosis until the date of the first occurrence of a new tumor event, which includes locoregional recurrence, distant metastasis, new primary tumor, or death with tumor. For a specific state, the progression-free survival probability was calculated using the progression-free interval and censoring information. Figure 2 depicts the Kaplan–Meier plots of progression-free survival of the seven states. We observed a clear trend of worsening survival functions along paths A, B, and C. Specifically, the clinical outcomes of the tumor samples in states 3, 4, and 6 were significantly worse than those in states 1, 2, and 5 [HR = 2.11 (1.40–3.19); *P* = 0.001]. It was noted that the survival outcomes cannot be predicted by the *TMPRSS2*–*ERG* fusion status, which is consistent with previous findings (35). Interestingly, we found that although the tumors in state 7 are located at the end of path D, the survival probability is similar to those of the upstream states (i.e., states 1 and 5) that are located at earlier stages of progression. This suggests that state 7 may represent slow-growing or dormant tumor subtypes.

**Figure 3:**
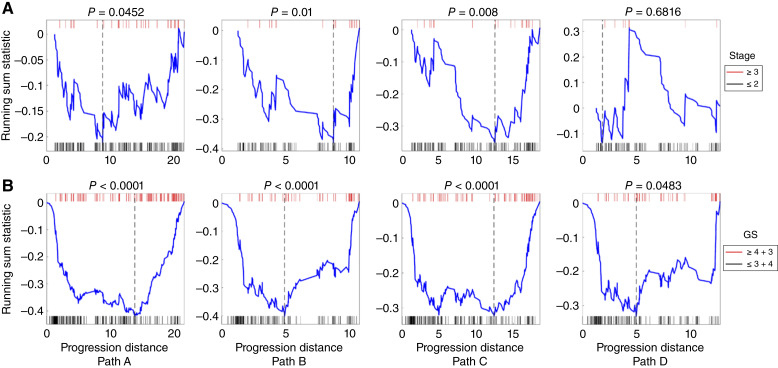
Enrichment analysis of T-stage and summary GS mapped onto four identified progression paths. **A,** T-stage. Tumor samples with T-stages 3 and 4 were plotted on the top (red), and those with T-stages 1 and 2 plotted at the bottom (black). **B,** Summary GS. Tumor samples with summary GS ≥ 4 + 3 were plotted on the top (red), and those with summary GS ≤ 3 + 4 plotted at the bottom (black). The *x*-axis represents the progression distances of tumor samples along a path, and *P* values were computed through 10,000 permutation tests. A negative enrichment score (defined as the maximum deviation of a running sum statistic from zero) indicates that T-stage ≥ 3 or GS ≥ 4 + 3 tumors were enriched at the end of a progression path, and a broken line indicates the location of the enrichment score on the progression path.

We next investigated the association between tumor stage (T-stage) and modeled progression paths. The T-stage is a measure of the size and extent of tissue invasion of a primary tumor, and a higher T-stage indicates a more malignant tumor (36). Accordingly, if the constructed model is valid, we would expect that low-stage and high-stage tumors are distributed at the early and later parts of the modeled progression paths, respectively. To this end, we mapped the stages of tumor samples back onto the progression model and performed a running sum enrichment analysis (see “Materials and Methods” for details; ref. 11). Figure 3A shows that high-stage tumors were significantly enriched at the later parts of paths A, B, and C (*P* < 0.05). However, no statistically significant association between increasing T-stage and the progression distance was revealed on path D. This result aligns well with path D/state 7 being comprised of a subset of tumors with favorable survival outcomes (Fig. 2).

We then mapped the GS onto each of the modeled progression paths. The GS is determined by a pathologist based on the microscopic appearance of prostate tissue available from biopsy or surgically excised material. The final GS is the summation of the primary and secondary grades present in a tissue specimen, with an increasing score being associated with a worse prognosis (13, 32, 37). The GS for each patient was mapped onto the model, and an enrichment analysis was performed (Fig. 3B). We found that high GS samples were significantly enriched toward the termini of all the four paths (path A: *P* < 1e–4, path B: *P* < 1e–4, path C: *P* < 1e–4, and path D: *P* = 0.0483). Notably, the relatively weak association for path D again supports the idea that state 7 represents a less aggressive disease state.

**Figure 4:**
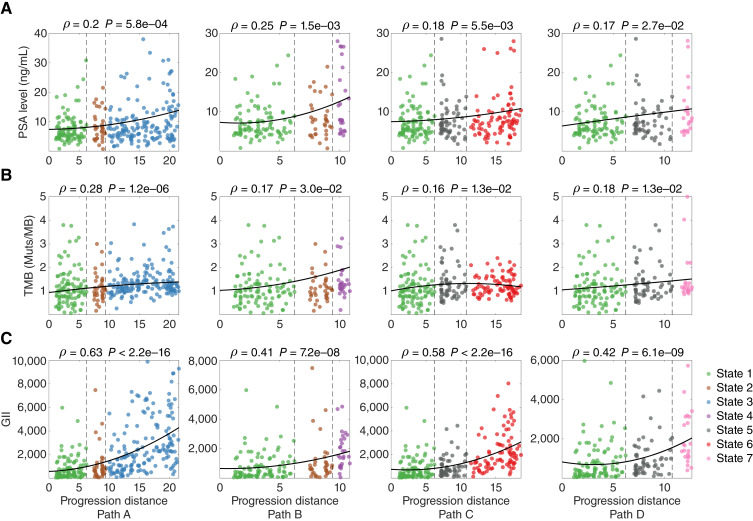
Spearman’s rank correlation analysis of PSA level, TMB, and GII mapped onto four identified progression paths. **A,** PSA level. **B,** TMB. **C,** GII. The broken line in each plot indicates the branching events on the corresponding progression path, and the solid line represents data trend estimated by natural spline curve fitting (df = 3).

We next investigated the association of the levels of preoperative PSA with progression. Elevated PSA levels in the blood are used as a biomarker for prostate cancer diagnosis and unfavorable prognosis (38–40). By mapping the preoperative PSA level (ng/mL) onto the model and evaluating its association with the progression distance by using Spearman’s rank correlation analysis, we observed positive correlations on all four progression paths (Fig. 4A; path A: *ρ* = 0.2, *P* = 5.8e–4, path B: *ρ* = 0.25, *P* = 1.5e–3, path C: *ρ* = 0.18, *P* = 5.5e–3, and path D: *ρ* = 0.17, *P* = 2.7e–2).

We finally performed an analysis by mapping onto the progression model two genetic variables, namely total mutation burden (TMB) and genome instability index (GII). TMB is defined as the number of somatic mutations per megabase in interrogated genomic sequences, and GII is defined as the summation of the magnitude of copy number alterations in all genes in a tumor specimen. It is recognized that cancer progression is associated with the accumulation of genomic alterations (11, 41–43), with mutations and copy number alterations playing a crucial role (44–47). Moreover, genome instability is considered an enabling characteristic of cancer evolution (48). Hence, TMB and GII can serve as the measures of tumor malignancy, and if the constructed model is valid, we would anticipate positive correlations between these variables and modeled progression distances. Notably, a significant correlation was observed between TMB and progression distance across all four paths (Fig. 4B; path A: *ρ* = 0.28, *P* = 1.2e–6, path B: *ρ* = 0.17, *P* = 3.0e–2, path C: *ρ* = 0.16, *P* = 1.3e–2, and path D: *ρ* = 0.18, *P* = 1.3e–2). Similarly, a strong correlation between GII and progression distance was observed on all four paths (Fig. 4C; path A: *ρ* = 0.63, *P* < 2.2e–16, path B:* ρ* = 0.41, *P* = 7.2e–8, path C: *ρ* = 0.58, *P* < 2.2e–16, and path D: *ρ* = 0.42, *P* = 6.1e–9). Overall, the described associations between a set of clinical and genetic variables with progression support the validity of the constructed model, which provides a foundation for the interrogation of dynamic molecular changes that drive prostate cancer progression.

### Identifying shared and distinct transcriptional signatures associated with disease progression

**Figure 5:**
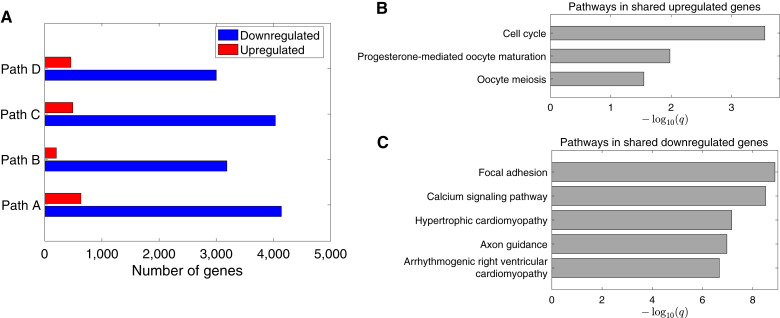
Transcriptional signatures identified in four progression paths. **A,** Numbers of upregulated and downregulated genes identified in each path. **B** and **C,** KEGG pathways enriched in the upregulated and downregulated genes shared by the four paths (*q*: FDR-adjusted *P* value).

We performed a transcriptional analysis to identify genes with expression levels that varied significantly along the modeled progression paths, with the aim to uncover important signaling pathways involved in the disease progression. Briefly, we identified genes by comparing a constant expression model with a nonlinear expression model using a likelihood-ratio test and controlled FDRs using the BH method (“Materials and Methods”). A total of 4,734, 3,389, 4,488, and 3,459 genes were detected for paths A–D, respectively (Supplementary Table S5). By grouping the identified genes into up- and downregulated modules (“Materials and Methods”), we found a much higher proportion of downregulated genes (Fig. 5A), which is consistent with previous findings that reported the predominant downregulation of molecular pathways in prostate cancer. We identified 136 genes commonly upregulated and 2,627 genes commonly downregulated across all four paths. To identify signaling pathways that were enriched in the detected genes, we performed a gene set enrichment analysis (“Materials and Methods”; ref. 29). We found that the shared upregulated genes were enriched in cell-cycle and oocyte meiosis pathways, whereas the shared downregulated genes were enriched in focal adhesion and calcium signaling pathways (Fig. 5B and C; Supplementary Table S6). The detected pathways are consistent with the established processes involved in prostate cancer tumorigenesis (10, 33, 49, 50).

**Figure 6:**
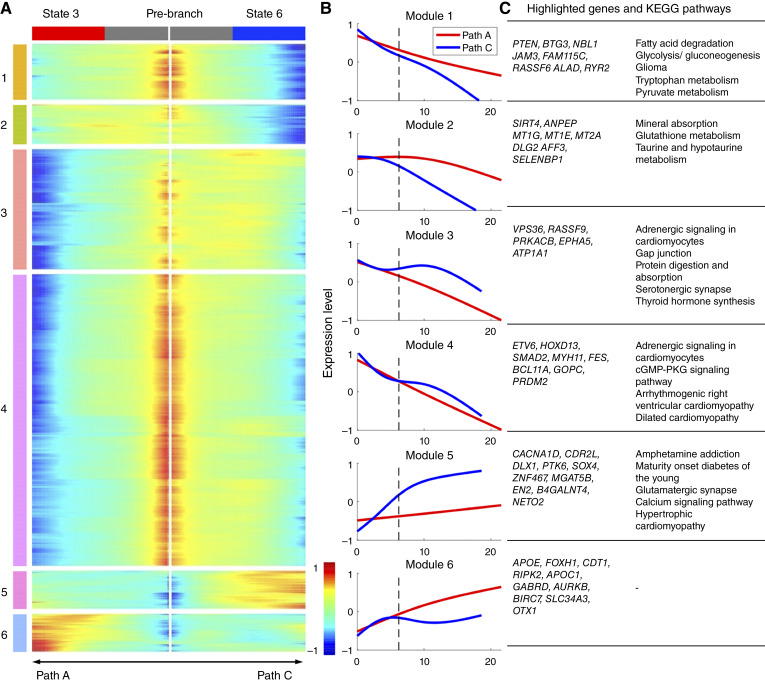
BEAM identified branch-dependent genes and molecular pathways associated with the divergence of *ERG* fusion–negative (path A) and *ERG* fusion–positive (path C) tumors. **A,** Heatmap of identified branch-dependent genes. The expression data of each gene were normalized into the range of (−1, 1) and smoothed by a natural spline function (df = 3). Based on expression patterns, the genes were clustered into six gene modules. **B,** Average expression curves of the six gene modules for the two paths. The broken lines indicate the branching events. **C,** Highlighted genes and KEGG pathways significantly enriched in each gene module. No pathways were identified to be significantly enriched in module 6.

We next sought to identify genes with distinct expression patterns along different disease progression paths exhibiting a branching point (indicated by the solid numbers in Fig. 1C). To this end, the BEAM (27) was employed. Briefly, we defined a null model that assumes identical gene expression patterns along two progression paths and an alternative model that assumes path-dependent patterns. Then, we detected genes by performing a likelihood-ratio test and corrected the test results for multiple comparisons (“Materials and Methods”). First, we focused on comparing progression signatures that diverged between *ERG* fusion–positive and *ERG* fusion–negative tumors, specifically those associated with path A (*ERG* fusion–negative) and path C (*ERG* fusion–positive). Applying the BEAM analysis to the 4,963 genes identified in at least one of the two paths, we detected 1,053 genes with distinct expression patterns between path A and path C. Based on expression patterns, these genes were clustered into six modules. Figure 6 depicts the heatmap (Fig. 6A), the average expression curves of the six gene modules (Fig. 6B) and highlighted genes/KEGG pathways (Fig. 6C), and Supplementary Tables S7 and S8 report a full list of prominent prostate cancer-related genes and the enriched KEGG signaling pathways for each module. Notably, modules 1 to 4 exhibited similar downregulation trends in both paths, whereas modules 5 to 6 showed contrasting expression trends. Modules 1 and 2 demonstrated earlier downregulation in path C compared with path A. These modules feature tumor suppressor genes *PTEN*, *BTG3*, and *SIRT4* (51–53) and genes associated with prostate cancer prognosis such as *NBL1* and *ANPEP* (54, 55). A number of genes associated with other cancers, including *JAM3*, *FAM115C*, *RASSF6*, *RYR2*, *MT1G*, *MT1E*, and *MT2A* (56–62), were also present in these modules. Conversely, modules 3 and 4 exhibited earlier downregulation in path A. These modules include genes *VPS36*, *RASSF9*, *PRKACB*, *ETV6*, and *HOXD13*, which have been reported to be downregulated in association with cancer progression and invasion (63–67). Module 5 exhibited unique upregulation in path C, the *ERG* fusion–positive branch, whereas remaining relatively stable in path A. This module includes *CDR2L*, *DLX1*, *SOX4*, and *ZNF467*, which have been reported to promote prostate cancer progression and invasiveness (68–71), and *CACNA1D*, a member of the voltage-gated calcium channel family, which can confer resistance to androgen deprivation therapy (ADT) in prostate cancer (72). Module 5 also includes a number of genes, including *MGAT5B*, *EN2*, *B4GALNT4*, and *NETO2*, which have not been associated with prostate cancer but have been implicated in other cancers (73–76). Module 6 was upregulated in path A and includes genes *APOE*, *FOXH1*, *CDT1*, and *RIPK2*, which have been reported to promote prostate cancer progression and metastasis (77–79).

The downregulation of tumor suppressor genes along the major paths is expected for the immortalization, increased genomic instability and mutagenesis, and the loss of apoptosis characteristic of nearly all cancers. For the *ERG* fusion–positive path C, several of the upregulated genes are transcription factors, including *SOX4* and *EN2*, which are involved in the regulation of development. This is consistent with the observation that *ERG*-positive tumors, unlike most other cancers, exhibit a more differentiated, less-plastic phenotype (80). Although this would suggest that *EGR*-positive tumors would be more responsive to ADT, surprisingly to date, this has not been found to be the case (81). On the other hand, *CACNA1D* was found to be selectively upregulated in path C, which could presumably provide a mechanism for these more differentiated cancer types to overcome hormonally regulated suppression of growth. It remains possible that further stratification of *ERG* fusion–positive tumors, possibly just due to *ERG* levels and/or in combination with lack of *CACNA1D* overexpression, might show different sensitivity to ADT. Conversely, for the *ERG*-negative path A, although there are also transcriptional regulators (e.g., *FOXH1* and *OTX1*), upregulation of factors involved in proliferation, such as *CDT1* and *AURKB*, and inhibition of apoptosis (*BIRC7*) are consistent with the proliferation and immortalization of cells characteristic of transformation (82, 83). Direct upregulation of such basal proliferation and immortalization factors is often consistent with less differentiated and more plastic cancer types that are often less responsive to hormonal regulation and more likely to result in poor prognosis (84, 85). Consistent with the progression modeling concept, the distal part of the two major branches, state 3 on path A (*ERG* fusion–negative) and state 6 on path C (*ERG* fusion–positive) had relatively worse survival rates (Fig. 2). This supports the idea that there are key late events that occur in the evolution of prostate cancer, regardless of *ERG* fusion status, that drive disease progression to a malignant phenotype (81, 82).

We then performed the BEAM analysis to identify genes that expressed differently between path A and path B. A total of 1,875 genes were detected, which were clustered into three modules (Supplementary Fig. S2A–S2C; Supplementary Tables S9 and S10). Notably, following the branching event, a decrease in the expression of the genes in each of the modules was observed in path A. Conversely, module 1 remained relatively unchanged along path B, but modules 2 and 3 showed an increase in expression. Module 1 includes the tumor suppressor genes *HOXD13*, *NTRK3*, and *SIX2* (67, 86, 87), and *RSPO3* and *STAT6*, which have been associated with prostate cancer prognosis (88, 89). Module 2 includes *COL3A1*, *COL5A2*, and *NOTCH3*, which have been reported to be associated with metastasis in prostate cancer (90–92), as well as *HEYL*, *STMN2*, and *ASPN*, which have been implicated in prostate cancer progression (93–95). Module 3 includes *NTRK2*, *DDR2*, and *STAT5B*, which have been associated with prostate tumor metastasis (96–98), and *JAZF1*, *ZEB1*, and *BCL2*, which have been linked to cancer progression and resistance to chemotherapy (99–101). The high expression of multiple genes associated with aggressive phenotypes suggests that path B/state 4 represents a subset of patients with poor prognosis and a high risk of metastasis, in alignment with the outcome data for state 4 (Fig. 2).

Finally, we performed the BEAM analysis of path C and path D. In total, we identified 1,352 genes, which were grouped into two expression modules (Supplementary Fig. S3A–S3C; Supplementary Tables S11 and S12). Again, the overall trend of gene expression was downregulation along path C, whereas module 1 expression was increased and module 2 expression was stable in path D. Module 1 includes *MSR1*, *THY1*, and *PILRA*, reported to be potential tumor suppressors (102–104), perhaps explaining, in part, why path D/state 7 represents a branch with a relatively good prognosis (Fig. 2). Module 2 exhibited relatively stable expression in path D but a sharp decrease in expression along path C (Supplementary Fig. S3B). Module 2 includes *TGFBR3*, *FOXO1*, *RSPO3*, *PLAG1*, and *CYLD*, which have been shown to play a role in mediating or inhibiting prostate cancer progression (88, 105–108).

The comparative analysis of progression paths, post-*ERG* fusion divergence, revealed key factors that are associated with disease progression regardless of the *ERG* fusion status. For example, *FOXO1* was markedly downregulated along both paths A and C in line with the increasingly poor outcome observed toward the termini of these progression paths. The ability to model progression and to pseudo-order tumor sample data onto the model facilitates the identification of key molecular events in the context of tumor evolution. In summary, our proposed progression model allowed for extensive analyses of shared and distinct transcription signatures across multiple progression paths. The divergent expression patterns that we discovered likely contribute to the separation of *ERG*-positive and *ERG*-negative samples in prostate cancer, as well as the formation of branching events within the two main branches. These findings provide valuable insights into the molecular heterogeneity and underlying mechanisms driving disease progression.

### Identifying oncogenetic events associated with prostate cancer progression

**Figure 7:**
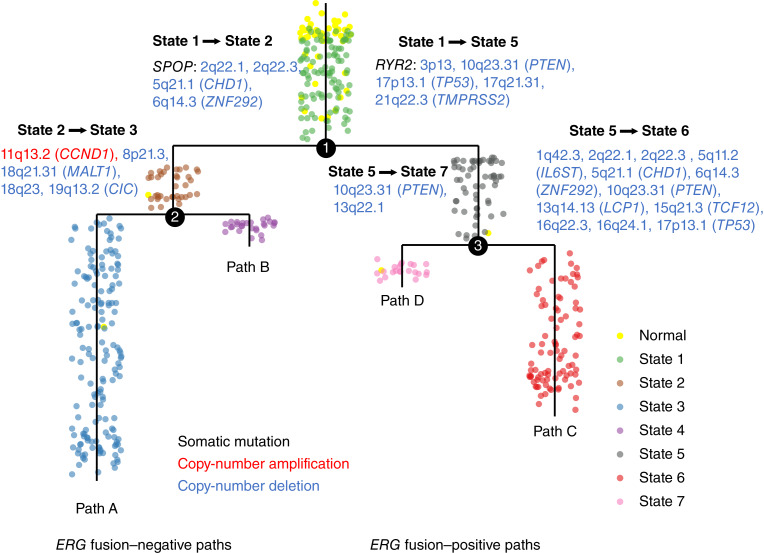
Putative oncogenetic events associated with transition between states on specific progression pathways. Black, somatic mutation; red, copy number amplification; and blue, copy number deletion. Within listed regions of CNV, genes previously identified to be associated with cancer were indicated in parentheses.

Identifying cancer driver genes is a central task of current cancer research (109–111). It can greatly advance our understanding of the genetic basis of tumorigenesis and provide actionable therapeutic targets. Due to the lack of cancer progression information, mainstay methods are prevalence-based approaches that work by grouping samples together and searching for genes that are mutated more frequently than random chance (112, 113). Consequently, they can only reveal the association between genetic events and disease in an overall sense. The development of a cancer progression model removes the above constraint, providing a unique opportunity to detect oncogenetic events associated with specific progression paths or states and put them in the context of a dynamic disease process. Here is the basic idea. After we constructed a progression model, we projected tumor samples back onto identified progression paths. If a genetic event confers malignant growth advantages and causes clonal expansion, by the cancer evolution theory (114), we would expect that the incidence rate of the genetic event is increased in advanced disease (115). In this study, we examined 21,225 nonsilent somatic mutations and copy number focal peaks including 28 amplifications and 35 deletions in 497 TCGA tumor samples. We performed a comparative analysis on each genetic event detected in two successive progression states to test whether the genetic event is more prevalent in the later state, and thus, it may play a role in driving cancer progression. The *P* values were computed by the right-tailed Fisher’s exact test (116), and the FDRs were controlled using the BH method (“Materials and Methods”). At the FDR level less than 1e-2, a total of 30 genetic events were detected, including two somatic mutations, one copy number amplification, and 28 copy number deletions (Fig. 7). The genes contained in each identified CNV focal peak are listed in Supplementary Table S13. We identified putative oncogenetic events in all disease state pairs, except for state 2 → state 4, potentially due to the limited number of samples in state 4 (27 samples, accounting for 4.9% of the cohort). We observed that the majority of the identified events were copy number deletions, which is consistent with previous findings (33). Notably, two detected somatic mutations, *SPOP* and *RYR2*, were associated with distinct early state transitions (*SPOP*: state 1 → state 2, and *RYR2*: state 1 → state 5), with the *ERG* fusion–negative (*SPOP*) and *ERG* fusion–positive (*RYR2*) branches exclusively.

During the early progression of *ERG* fusion–negative tumors, *SPOP* mutations were associated with deletions of 2q22.1, 2q22.3, 5q21.1 (*CHD1*), and 6q14.3 (*ZNF292*). The co-occurrence of *CHD1* deletion and *SPOP* mutation in *ERG* fusion–negative prostate cancer has been described previously (117), but progression modeling reveals these as early, codriving events. The deletion of the tumor suppressor *ZNF292* on 6q14.3 also seems to be an early driving event in *ERG* fusion–negative tumors (118, 119). The subsequent transition from state 2 to state 3 in *ERG* fusion–negative tumors was characterized by the amplification of 11q13.2 (*CCND1*) and deletions of 8p21.3, 18q21.31 (*MALT1*), 18q23, and 19q13.2 (*CIC*). The amplification of the 11q13.2 region has been associated with aggressive disease in various cancer types, including prostate (120, 121), and 18q21.31 and 18q23 deletions have been linked to poor clinical outcomes in prostate tumors (122, 123). No specific genetic events were associated with transition from state 2 to state 4 in *ERG* fusion–negative tumors.

Conversely, transition from state 1 to state 5 on the *ERG* fusion–positive path *RYR2* mutation was associated with deletions of 3p13, 10q23.31 (*PTEN*), 17p13.1 (*TP53*), 17q21.31, and 21q22.3 (*TMPRSS2*). The *RYR2* gene product regulates calcium signaling and can inhibit apoptosis (124–127). The deletion of 21q22.3 is the result of the observed *TMPRSS2*–*ERG* fusion event (128, 129), and the loss of the tumor suppressor gene *PTEN* at 10q23.31 is known as a key driving event in cancer. The deletion of *TP53* at 17p13.1 (46, 130) was the most prevalent genetic event on path C, and along with *PTEN* loss, occurred at multiple transition points (state 1 to 5 and 5 to 6). The largest number of genetic events occurred during the transition from state 5 to state 6. Deletions in 1q42.3, 2q22.1, 2q22.3, 5q11.2 (*IL6ST*), 13q14.13 (*LCP1*), 15q21.3 (*TCF12*), 16q22.3, and 16q24.1 were specific to this transition, whereas deletions in 5q21.1 (*CHD1*), 6q14.3 (*ZNF292*) were also identified in the *ERG* fusion–negative progression path. The accumulation of multiple deletions involving known cancer driver genes in state 6 may explain the poor prognosis associated with this subset of tumors. The identification of deletions in *CHD1* and *ZNF292* in both path A and path C suggests a key role for these genes in disease progression *regardless* of *ERG* fusion status. Finally, two deletions [10q23.31 (*PTEN*) and 13q22.1] were associated with the transition from state 5 to state 7 in *ERG* fusion–positive tumors. The 13q22.1 focus has not been extensively investigated in prostate cancer, but it has been noted to be deleted in pancreatic and colon cancers (131–133).

### Validating the constructed model using an independent dataset

**Figure 8:**
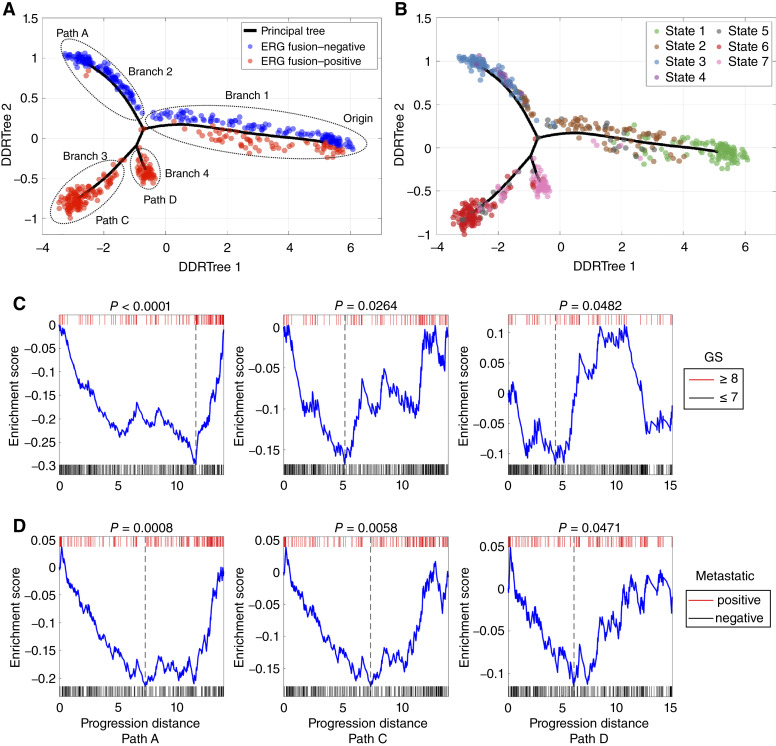
Progression modeling analysis performed on an independent prostate cancer dataset and validation of the constructed model. **A,** Visualization analysis showed the tumor sample distribution in the space spanned by the two leading components (denoted as DDRTree 1–2) generated using the DDRTree algorithm. The black line represents the constructed principal tree. Each tumor sample was color-coded by its *ERG* fusion status inferred by the *ERG* gene expression level. Three progression paths were identified, referred to as paths A, C, and D, respectively. **B,** Tumor samples were color-coded by seven progression states identified in the TCGA model. **C** and **D,** Enrichment analysis of GS and metastatic status of tumor samples mapped onto the three progression paths identified in the constructed model. The *x*-axis represents the progression distances of tumor samples, and *P* values were calculated through 10,000 permutation tests.

To further investigate the validity of the constructed model, we performed a *de novo* progression modeling analysis to demonstrate that a similar progression structure can be repeatedly observed in an independent dataset. We believe that this is probably the most rigorous way one could use for model validation. In the process, we did not rely on any information from the original dataset. Furthermore, because the new dataset was processed independently, the constructed model is free from any possible biases inherent in the original model. The validation dataset, comprised of gene expression data from 545 primary prostate tumor samples, was downloaded from GEO (GEO accession number: GSE46691; ref. 134). By using the same modeling approach, we identified 58 genes for modeling analysis (Supplementary Table S14) and constructed a model that was referred to as the GSE model (Fig. 8). Due to the lack of data from the normal tissue samples in the GSE data, we were not able to determine the origin of disease progression directly. To address this issue, we inferred the progression origin and aligned the GSE model with the TCGA model (“Materials and Methods”). The previous work (33) and our TCGA model have indicated that *ERG* fusion–positive and –negative prostate cancer are developed from normal prostate gland independently and mutually exclusively. Hence, the progression origin could be inferred indirectly based on the *ERG* fusion status. However, the GSE data did not contain *ERG* fusion information. Because the expression of *ERG* in prostate tumor tissue is primarily driven by the *TMPRSS2*–*ERG* fusion event (135), we inferred the *ERG* fusion status based on the expression level of the *ERG* gene (Fig. 8A). Interestingly, as with the TCGA model, one main branch of the GSE model (i.e., branch 1) contained a mix of *ERG* fusion–positive and –negative tumor samples. We thus speculated that the starting point of branch 1 was the origin of disease progression. To confirm this, we developed a *k*-nearest neighbors classifier using the TCGA data and classified each sample in the GSE data as one of the seven states identified in the TCGA model (“Materials and Methods”; Fig. 8B). We found that branch 1 was highly enriched with state 1 samples (*P* = 1.4e–7, binomial test), supporting the idea that branch 1 contains the earliest state in the TCGA model (see Fig. 1C). We also found that branches 2, 3, and 4 were highly enriched with the samples classified as state 3 (*P* = 5.3e–8), state 6 (*P* = 6.7e–12), and state 7 (*P* = 1.3e–8), respectively. Following the annotation used in the TCGA model, we extracted three progression paths and named them as paths A, C, and D correspondingly (Fig. 8A; Supplementary Table S15).

The topologic structure of the GSE model is very similar to that of the TCGA model. Starting from normal or a branch that contained a mixture of *ERG* fusion–positive and –negative samples, both models displayed a linear, bifurcating progression pattern with two major branches associated almost exclusively with *ERG* fusion–positive or –negative tumor samples. The GSE model also revealed a side branch on the *ERG*-positive path, as observed in the TCGA model (state 7, path D), but no side branch was identified on the *ERG*-negative path. The discrepancy may be attributed to various factors, including sampling bias, the utilization of different platforms (RNAseq vs. microarray), and cohort composition.

As with the analysis that we performed on the TCGA data, we mapped the clinical variables onto the GSE model and performed enrichment tests (Fig. 8C and D). Although there were considerably fewer clinical data available in the GSE data compared with the TCGA data, significant associations between the progression distance and T-stage (metastatic status) and GS were detected on all three modeled progression paths.

In addition to the *de novo* analysis, we also performed a progression modeling analysis by mapping the GSE data onto the TCGA data (see Supplementary Data for details). We found that the structure of the obtained model is very similar to that of the TCGA model, which provided further support for the proposed model (Supplementary Fig. S4).

## Discussion

Prostate cancer is a clinically heterogeneous disease, and this is reflected in the diverse molecular patterns observed in prostate tumor tissues. Studies from the TCGA consortium have provided a detailed molecular taxonomy of primary prostate cancers (33), but the identification of fewer prevalent molecular changes and how the observed molecular profiles align with progression from early to late-stage disease remains a challenge. It is now widely recognized that malignant tumors are comprised of multiple subclonal populations that evolve over time. Insights into the evolutionary process have been fueled by advances in molecular profiling technologies and in computational biology, and identification of the processes that drive disease progression is beginning to emerge. Here, we leveraged the TCGA RNAseq data to construct a model of prostate cancer progression. The computational strategy is based on our previous work whereby pseudo time series data are derived from static samples (i.e., single timepoint data obtained from excised tumor tissue samples; ref. 11). The modeling approach sequentially involves feature selection, principal curve learning, data visualization, model construction, and progression path extraction. The progression model provides a framework for the identification of key molecular changes and distinct molecular states or subtypes in the context of tumor evolution.

The TCGA model revealed four major progression paths from a baseline of normal prostate tissue samples. The first major bifurcation point was dominated by the presence, or absence, of fusion events involving members of the *ETS* family, primarily the fusion of *TMPRSS2*–*ERG*. This separation in modeled space supports a role for *ERG* fusion detection in prostate tumor classification, and the visualization of *ERG* fusion–positive samples within the earliest branch of the model (state 1) confirms that the *ERG* fusion event occurs very early in prostate tumor initiation or development. Each of the resulting major progression arms had a secondary branch point with a major and minor component. By partitioning samples based on model topology, we identified seven states or subtypes that can be considered as parts of this progressive multi-branched series. Comparative analyses between progression paths and between progression states along a path identified genetic and transcriptomic features associated with the transition toward more aggressive disease. Support for the validity of the constructed model was derived by the expected correlation of patient outcome, GS, T-stage, and blood PSA level, plus the accumulation of DNA mutation burden and genome stability index with progression distances. Furthermore, the application of the same modeling approach to an independent prostate cancer dataset revealed a markedly similar model structure, validating the obtained model generated using the TCGA dataset.

Although the spectrum of mutations and copy number alterations in prostate tumors has been previously characterized (33), by ordering tumor sample data in a progression model, we were able to put these into the context of cancer progression and uncover differences associated with specific evolutionary trajectories and transition between progressive states. The molecular profiles that define these disease states may be leveraged to derive molecular subtypes for use in diagnosis, prognosis, or treatment monitoring. The tracking of molecular changes along distinct progression paths revealed both common and divergent genetic and transcriptional signatures associated with advancing disease. The modeling confirmed the distinction between tumors with *ETS* fusions and identified this as one of the earliest events in disease development (10, 33), but for both *ERG*-positive and *ERG*-negative phenotypes, there are also other key early events involved in transition to the intermediate progression state. When visualized as a linear model, we observed potential driving events in both *ERG*-positive and *ERG*-negative tumors, but at different stages of progression. For example, loss of *CHD1* and *ZNF292* occurs at a later stage in *ERG*-positive tumors than in *ERG* fusion–negative tumors, but as these lesions are common to both progression pathways, these genes seem to be key drivers of prostate cancer progression. Another example is the implication of *RYR2* mutations as an early event in a subgroup of *ERG*-positive tumors. *RYR2* mutations have previously been identified in several cancers (124–127), but through comparative analysis guided by progression modeling, it was revealed as an important event in prostate cancer. Conversely, the loss of *PTEN* and *TP53* via genetic events was revealed to occur at multiple points of transition and progression, confirming these factors as key compounding events in advancing disease (51, 52).

Our findings in prostate cancer progression modeling also have clinical implications. The early discrimination of the *SPOP* mutant from *ERG* fusion–positive lineage and the previous demonstration that *SPOP* mutant cancers are sensitive to ADT , whereas *ERG* overexpressing lines are resistant (136), are consistent with our results. These findings support the potential role of prostate cancer tumor lineages in predicting the candidacy for ADT. Moreover, we found that tumors in state 7 on path D had high probability of progression-free survival and low prevalence of metastatic disease. Potentially, patients who present with the *ERG*–*TMPRSS2* fusion, but also show characteristics of the transition to path D (characterized by upregulation of *MSR1*, *THY1*, *PILRA*, *BGN*, and *FAP*), would be candidates for active surveillance, a tailored clinical management plan for the slow-growing, often indolent prostate cancer. Further studies in these directions are warranted.

A limitation of our study is the reliance on data obtained from bulk tissue homogenization methods, which do not provide information on intratumor heterogeneity or disease multifocality prevalent in prostate cancer. The progression modeling approach can be performed using multiple sources of data, so as single-cell and spatial biology approaches become mainstream (137, 138), data from more precise tumor and microenvironment components can be incorporated into the modeling approach. We are continuing to refine the computational methods to provide an increasingly accurate and efficient progression model pipeline. Once a progression model is established, new individual cases can be mapped onto the model for the evaluation of disease status, while providing cumulative data for model interrogation.

In summary, our study provides a new perspective on prostate disease progression. The ability to use static sample data to construct progression models facilitates the delineation of dynamic disease processes and the identification of pivotal molecular events that drive stepwise disease progression. The annotation of progressive changes into a prostate cancer development roadmap can support the development of improved subtyping and biomarker development and potentially identify additional therapeutic targets. Future studies in preclinical models and in clinical trial settings will be required to confirm the translational potential of identified molecular changes associated with disease progression, but appropriate models will undoubtably provide important guides and references for such studies and offer insights for clinical management of prostate cancer.

## Supplementary Material

Supplementary Tables S2-4 TCGA disease-related genes, states and paths.

Supplementary Tables S5-12 Significantly varied genes, BEAM results, enriched pathways.

Supplementary Table S13 Putative driver genetic events in transitions.

Supplementary Tables S14-15 GSE46691 disease-related genes, paths.

Figure S1 Supplementary Figure S1

Figure S2 Supplementary Figure S2

Figure S3 Supplementary Figure S3

Figure S4 Supplementary Figure S4

Supplementary Data Construction of combined model.

Supplementary Table S1 Data, methods and softwares used.
