# Supplementary material for: Prostate Cancer Progression Modeling Provides Insight into Dynamic Molecular Changes Associated with Progressive Disease States
Source: Cancer Res Commun. 2024 Oct 24;4(10):2783–98. doi: 10.1158/2767-9764.CRC-24-0210 (PMC11500312; doi:10.1158/2767-9764.CRC-24-0210)
Supplement: Figure S1 — Supplementary Figure S1 [file crc-24-0210_figure_s1_suppsf1.pdf]

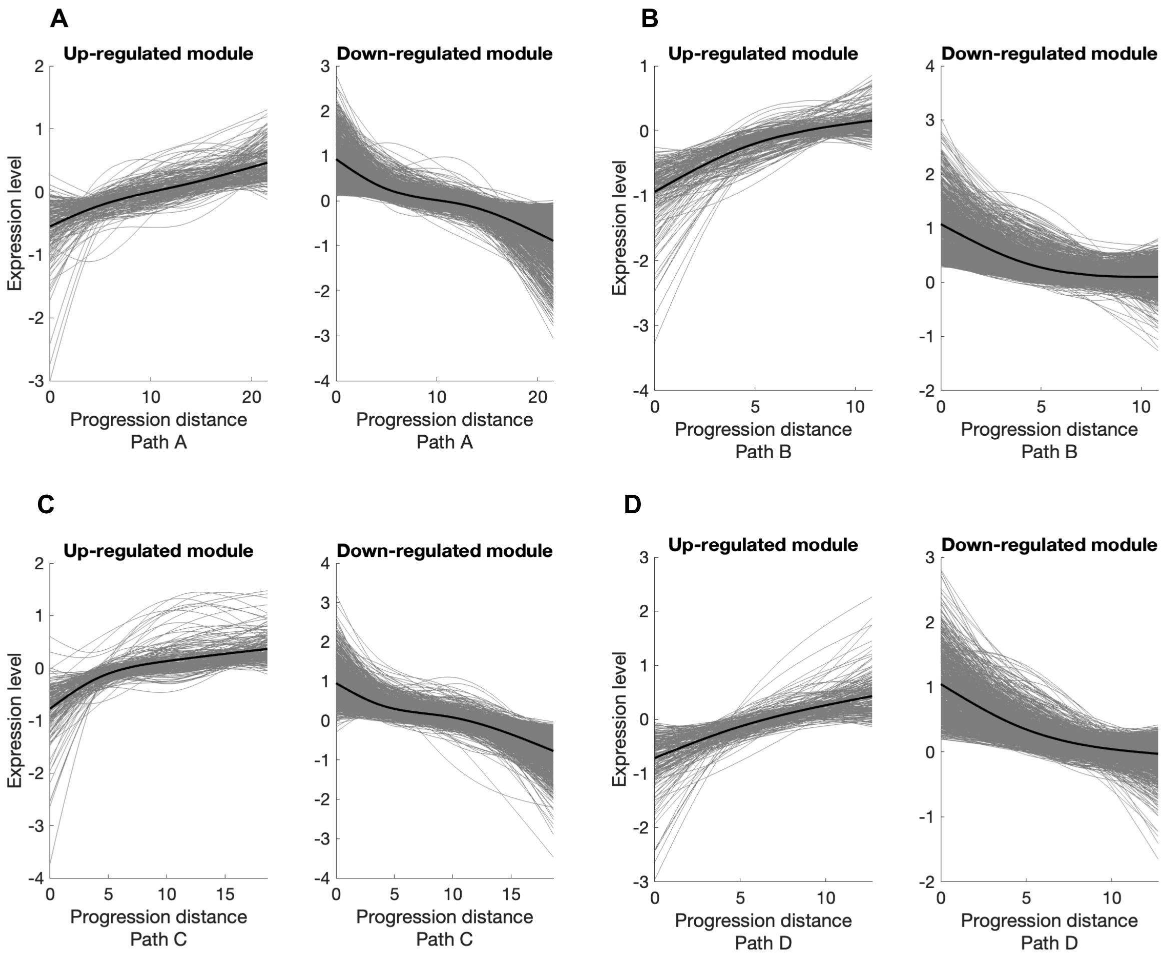

Figure S1: Genes with expression levels that changed significantly along four progression paths identified in the TCGA model were clustered into an up-regulated and a down-regulated gene module by using the *k*-medois algorithm. (A) Path A. (B) Path B. (C) Path C. (D) Path D. Each line represents a gene curve generated by fitting a natural spline function to the expression data of a gene.
