# Supplementary material for: Prostate Cancer Progression Modeling Provides Insight into Dynamic Molecular Changes Associated with Progressive Disease States
Source: Cancer Res Commun. 2024 Oct 24;4(10):2783–98. doi: 10.1158/2767-9764.CRC-24-0210 (PMC11500312; doi:10.1158/2767-9764.CRC-24-0210)
Supplement: Figure S2 — Supplementary Figure S2 [file crc-24-0210_figure_s2_suppsf2.pdf]

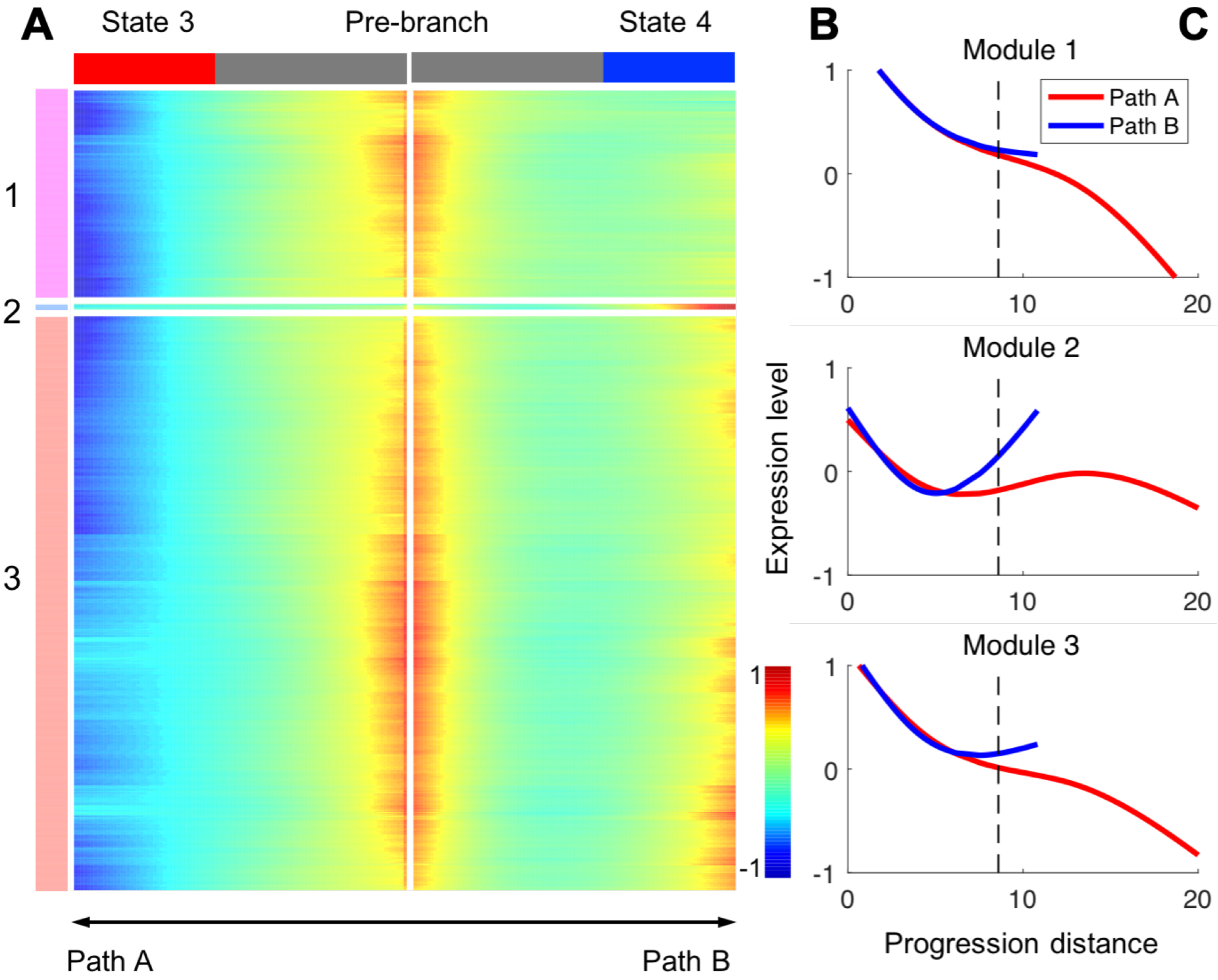

C

Highlighted genes and KEGG pathways	
HOXD13, NTRK3 SIX2 RSPO3, STAT6	Arrhythmogenic right ventricular cardiomyopathy<br>Dilated cardiomyopathy<br>Axon guidance<br>Hypertrophic cardiomyopathy<br>ECM-receptor interaction
COL3A1, COL5A2 NOTCH3, HEYL STMN2, ASPN	-
DDR2, FOXO1 STAT5B, ZEB1, FLNA, NTRK2 JAZF1, PRRX1, PRDM2, BCL2, PML	Focal adhesion<br>Proteoglycans in cancer<br>Oxytocin signaling pathway<br>Calcium signaling pathway<br>cGMP-PKG signaling pathway

Figure S2: Branch expression analysis modeling identified branch-dependent genes and molecular pathways associated with the divergence of Path A and Path B tumors. (A) Heatmap of identified branch-dependent genes, which were clustered into three gene modules. (B) Average expression curves of the three gene modules for the two paths. The broken lines indicate the branching events. (C) Highlighted genes and KEGG pathways significantly enriched in each gene module. No pathways were identified to be significantly enriched in Module 2.
