# Supplementary material for: Prostate Cancer Progression Modeling Provides Insight into Dynamic Molecular Changes Associated with Progressive Disease States
Source: Cancer Res Commun. 2024 Oct 24;4(10):2783–98. doi: 10.1158/2767-9764.CRC-24-0210 (PMC11500312; doi:10.1158/2767-9764.CRC-24-0210)
Supplement: Figure S3 — Supplementary Figure S3 [file crc-24-0210_figure_s3_suppsf3.pdf]

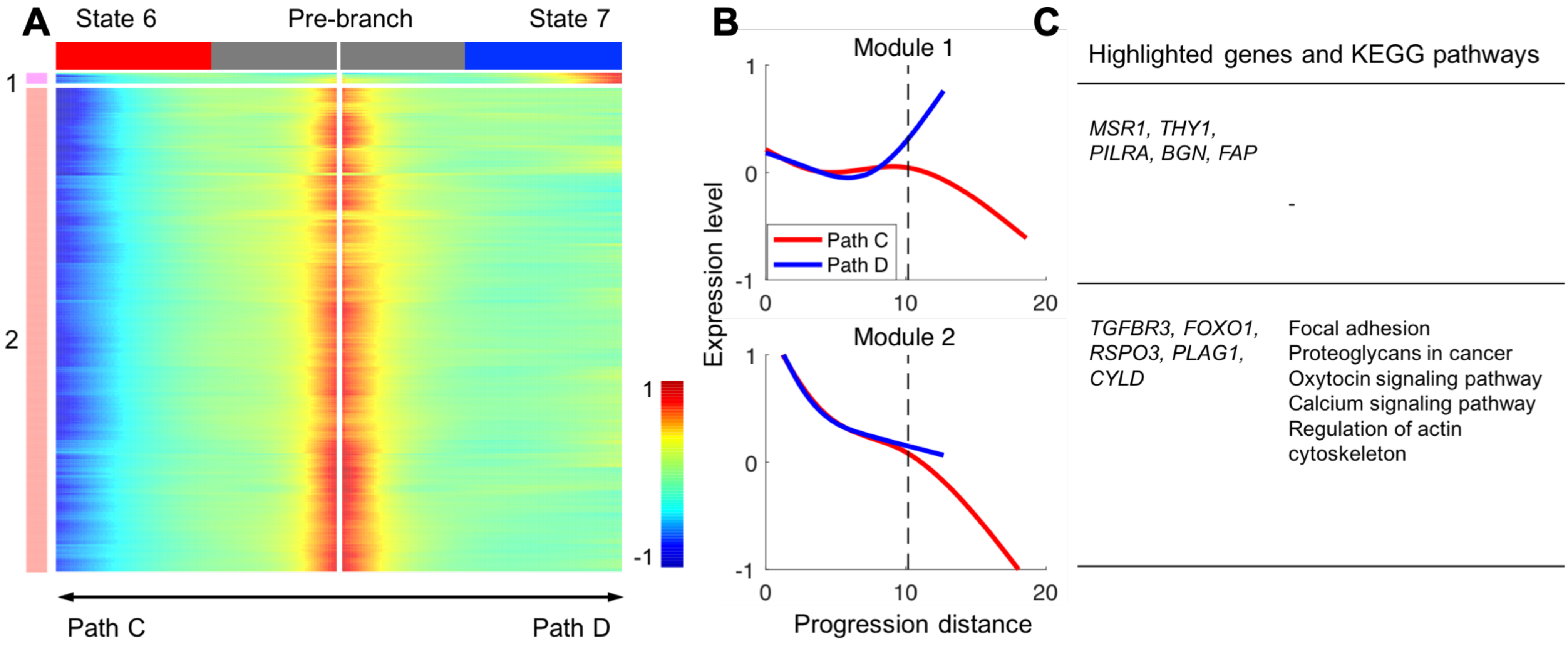

Figure S3: Branch expression analysis modeling identified branch-dependent genes and molecular pathways associated with the divergence of Path C and Path D tumors. (A) Heatmap of identified branch-dependent genes, which were clustered into two gene modules. (B) Average expression curves of the two gene modules along the two paths. The broken lines indicate the branching events. (C) Highlighted genes and KEGG pathways significantly enriched in each gene module. No pathways were identified to be significantly enriched in Module 1.
