# Supplementary material for: Prostate Cancer Progression Modeling Provides Insight into Dynamic Molecular Changes Associated with Progressive Disease States
Source: Cancer Res Commun. 2024 Oct 24;4(10):2783–98. doi: 10.1158/2767-9764.CRC-24-0210 (PMC11500312; doi:10.1158/2767-9764.CRC-24-0210)
Supplement: Figure S4 — Supplementary Figure S4 [file crc-24-0210_figure_s4_suppsf4.pdf]

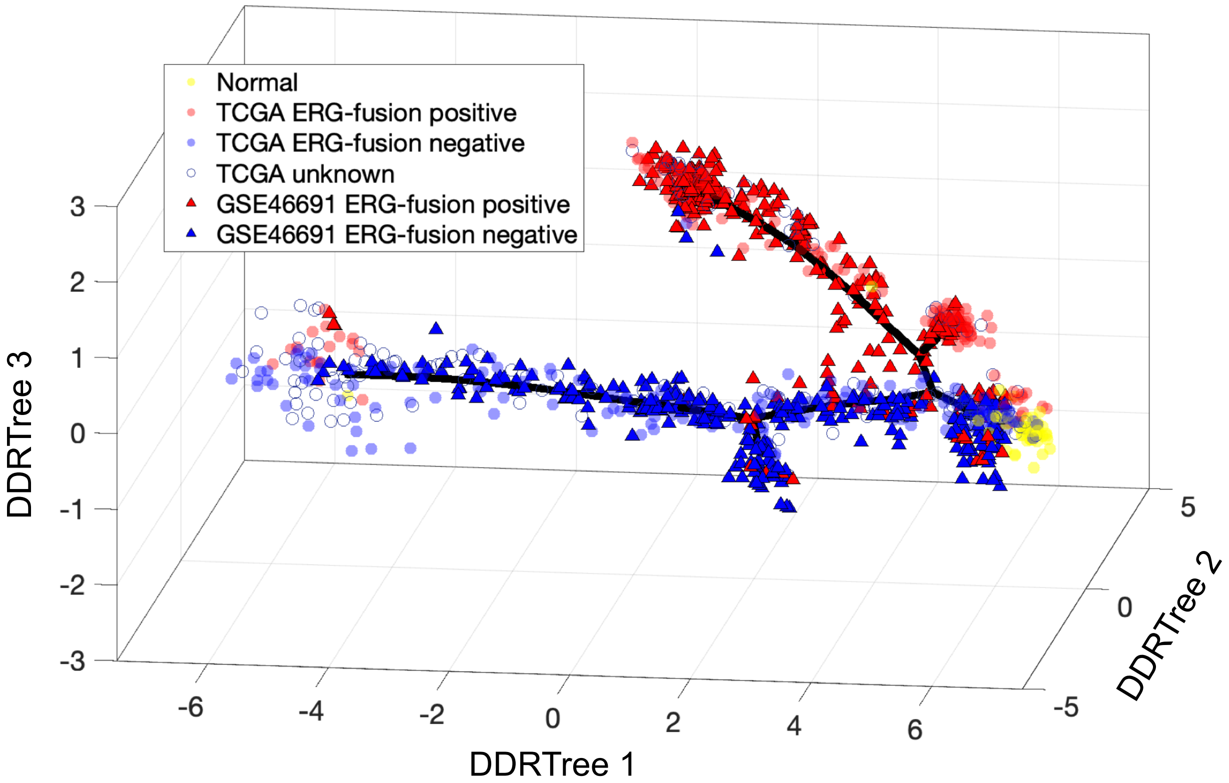

Figure S4: Progression modeling analysis performed on the combined TCGA and GSE dataset. The black line represents the constructed principal tree. Each circle or triangle presents a tumor sample, color-coded by its *ERG*-fusion status.
