# Supplementary material for: Prostate Cancer Progression Modeling Provides Insight into Dynamic Molecular Changes Associated with Progressive Disease States
Source: Cancer Res Commun. 2024 Oct 24;4(10):2783–98. doi: 10.1158/2767-9764.CRC-24-0210 (PMC11500312; doi:10.1158/2767-9764.CRC-24-0210)
Supplement: Supplementary Data — Construction of combined model. [file crc-24-0210_supplementary_data_suppsd.pdf]

Supplementary Data

Progression Modeling Analysis Using Combined Data

Due to batch effect and other issues (for example, genes covered by different studies may only be partially overlapped), data from different studies may not be directly comparable. Nevertheless, we performed an additional progression modeling analysis by combining the TCGA and GSE datasets. Specifically, we first mapped the 43 disease-related genes identified in the TCGA data to the GSE data and identified 40 common genes. Then, we applied ComBat [1] to the expression data of the common genes to remove the platform-induced batch effect in the two datasets. Finally, we applied the DDRTree method to the combined dataset and performed principal curve learning and dimension reduction. Figure S4 presents the sample distribution of the combined TCGA and GSE datasets, where each sample was color-coded by its *ERG*-fusion status. We can see that the obtained model closely resembles the TCGA model, featuring two main branches and sub-branches on each main branch. The distribution of the GSE samples confirms that in the independent dataset, the main branches of *ERG*-fusion positive and negative samples, as well as the sub-branches on both main branches, are also present.

References

[1] Johnson, W. E., Li, C., and Rabinovic, A. (2007) Adjusting batch effects in microarray expression data using empirical Bayes methods. *Biostatistics,* **8**(1), 118–127.
